# BRD4 Targets the KEAP1-Nrf2-G6PD Axis and Suppresses Redox Metabolism in Small Cell Lung Cancer

**DOI:** 10.3390/antiox11040661

**Published:** 2022-03-29

**Authors:** Yang Lv, Xiaotong Lv, Jiahui Zhang, Guozhen Cao, Changzhi Xu, Buchang Zhang, Wenchu Lin

**Affiliations:** 1 High Magnetic Field Laboratory, Hefei Institutes of Physical Science, Chinese Academy of Sciences, Hefei 230031, China; zwscjmx@mail.ustc.edu.cn (Y.L.); lvxt@mail.ustc.edu.cn (X.L.); zjhui@mail.ustc.edu.cn (J.Z.); cgzhwaxx@mail.ustc.edu.cn (G.C.); 2 University of Science and Technology of China, Hefei 230026, China; 3 Key Laboratory of High Magnetic Field and Ion Beam Physical Biology, Hefei Institutes of Physical Science, Chinese Academy of Sciences, Hefei 230031, China; 4 Institutes of Physical Science and Information Technology, Anhui University, Hefei 230601, China; xcz@ahu.edu.cn (C.X.); zhbc@ahu.edu.cn (B.Z.)

**Keywords:** small cell lung cancer, BRD4, KEAP1, Nrf2, pentose phosphate pathway

## Abstract

Accumulating evidence has witnessed the Kelch-like ECH-associated protein 1(KEAP1)- nuclear factor (erythroid-derived 2)-like 2 (Nrf2) axis is the main regulatory factor of cell resistance to endogenous and exogenous oxidative assaults. However, there are few studies addressing the upstream regulatory factors of KEAP1. Herein, bioinformatic analysis suggests bromodomain-containing protein 4 (BRD4) as a potential top transcriptional regulator of KEAP1 in lung cancer. Using molecular and pharmacological approaches, we then discovered that BRD4 can directly bind to the promoter of *KEAP1* to activate its transcription and down-regulate the stability of Nrf2 which in turn transcriptionally suppresses glucose-6-phosphate dehydrogenase (G6PD) in small cell lung cancer (SCLC), a highly proliferative and aggressive disease with limited treatment options. In addition, BRD4 could associate with the Nrf2 protein in a non-KEAP1-dependent manner to inhibit Nrf2 activity. Furthermore, simultaneous application of JQ1 and ATRA or RRx-001 yielded synergistic inhibition both in vitro and in vivo. These data suggest metabolic reprogramming by JQ1 treatment improves cell resistance to oxidative stress and might be a resistance mechanism to bromodomain and extra-terminal domain (BET) inhibition therapy. Altogether, our findings provide novel insight into the transcriptional regulatory network of BRD4 and KEAP1 and transcriptional regulation of the pentose phosphate pathway in SCLC.

## 1. Introduction

Redox imbalance with increased generation of reactive oxygen species (ROS) has been implicated in the pathogenesis of diverse disease conditions including cancer. The Kelch-like ECH-associated protein 1(KEAP1)- nuclear factor (erythroid-derived 2)-like 2 (Nrf2) pathway is the primary regulator of cellular protection response to endogenous and exogenous threats caused by ROS and electrophiles [1]. The inherent reactivity of the thiol group of cysteines on KEAP1 could sense redox assaults and allow Nrf2 release from KEAP1 to activate a battery of anti-oxidative genes. A growing number of studies have recorded abnormalities in the KEAP1-Nrf2 system under pathological conditions including *KEAP1* and *Nrf2* mutations, DNA hypermethylation in the *KEAP1* promoter region, and *KEAP1* gene heterozygotic deletion [2,3]. Moreover, extensive studies have documented the importance of the proper interplay between *KEAP1* and *Nrf2* for various biological events. However, transcriptional regulations of *KEAP1* expression, especially in an epigenetic manner, remain largely unexplored.

Glucose, a key intracellular fuel, is metabolized by glycolysis to produce nicotinamide adenine dinucleotide phosphate (NADPH), besides ATP and biomass, which is required to scavenge ROS via the oxidative pentose phosphate pathway (PPP) [4,5]. In the past decades, accumulating data have indicated that the flux of the PPP is increased in response to the tumor microenvironment and intracellular demands to promote cancer cell proliferation and survival across cancers [6,7]. Regulation of the PPP pathway entails a myriad of different aspects. For instance, the expression and activity of glucose-6-phosphate dehydrogenase (G6PD), a rate-limited enzyme in the oxidative PPP pathway, is tightly regulated by several oncoproteins and tumor suppressors [8,9]. In addition, several recent studies have shown the importance of the interplay between G6PD and the KEAP1-Nrf2 axis. Nrf2 up-regulation could enhance *G6PD* expression, promoting the proliferation and migration of breast cancer cells [10]. Constitutive activation of Nrf2 by inactivating mutations in *KEAP1* or activating mutations in *Nrf2* interferes with the KEAP1-Nrf2 interaction, reprogramming glucose metabolism to support cell proliferation and contribute to cancer progression [11,12]. Furthermore, sustained activation of Nrf2 can up-regulate the expression of the PPP genes by attenuating miR-1 and miR-206 expression, enhancing PPP-dependent NADPH production and promoting tumor cell growth [11,13]. However, how the KEAP1-Nrf2 axis modulates G6PD and the PPP pathway has yet to be fully characterized.

The bromodomain and extra-terminal domain (BET) family members have been recognized as potential therapeutic targets across human cancers. Specifically, BET proteins include bromine domain proteins 2, 3, and 4 (BRD2, BRD3, and BRD4) and testicular-specific bromine domain proteins (BRDT) [14]. Bromodomain-containing protein 4 (BRD4) is the most widely studied BET member and is up-regulated in a broad spectrum of human cancers. For example, high levels of BRD4 are closely associated with poor prognosis in patients with non-small-cell lung cancer (NSCLC) [15] and breast cancer [16]. Based on the encouraging anti-tumor activity of BRD4 inhibition in hematological malignancies and solid tumors such as prostate cancer [17], glioblastoma [18], neuroblastoma [19], and lung cancer [20], a number of BRD4 inhibitors have entered clinical trials for cancer treatment [21]. However, accumulating evidence has shown that intrinsic and acquired drug resistance occurs once applying BRD4 inhibitors in therapeutics [22]. For example, cancer cells can gain resistance to BET inhibitors (BETis) through adaptive kinome reprogramming, which means that JQ1 as monotherapy may not provide a lasting therapeutic response [23,24]. Therefore, it is critical to dissect the underlying mechanisms responsible for the resistance to improve the therapeutic efficacy of BETi.

This study employed bioinformatics and molecular approaches to explore the potential transcription factors directly activating *KEAP1* expression. We identified BRD4 as a potential factor positively modulating *KEAP1* expression at the transcriptional level in lung cancer. Using small cell lung cancer (SCLC) as a model, we further found that BRD4 binds to the promoter region of *KEAP1* and transcriptionally regulates its expression. Both knockdown of BRD4 by siRNA and JQ1 treatment dramatically down-regulated *KEAP1* expression, stabilized Nrf2, and activated its downstream targets. Importantly, we demonstrated that BRD4 could suppress G6PD through the KEAP1-Nrf2 axis. Finally, the combined use of JQ1 and ATRA or RRx-001 achieved synergistic anti-tumor activity in cellular and xenograft models based on the discovery of the novel mechanism. Our results contribute insight into the transcriptional regulatory network of BRD4 and nominate new and effective combination therapy strategies in treating SCLC, a poorly differentiated neuroendocrine cancer, and the most malignant type of lung cancer [25].

## 2. Materials and Methods

### 2.1. Cell Culture and Small Compounds

Human small cell lung cancer cells (H82, SHP77, H526, H69, and DMS273) were kindly provided by Dr. Matthew Meyerson’s Laboratory at Dana-Farber Cancer Institute, USA. H82, SHP77, H526, H69 are originally purchased from the American Type Culture Collection (ATCC) and DMS273 was originally obtained from the European Collection of Cell Cultures (ECACC). 

All human small cell lung cancer cells (H82, SHP77, H526, H69, and DMS273) were cultured at 37 °C in a humid atmosphere containing 5% carbon dioxide and in RPMI 1640 medium supplemented with 10% fetal bovine serum (FBS). JQ1 was purchased from Selleck Chemical (Shanghai, China), and acetylcysteine (NAC), ATRA, and RRx-001 were obtained from MedChemExpress (Shanghai, China). JQ1, ATRA, and RRx-001 were dissolved and aliquoted in DMSO (Sigma-Aldrich, Shanghai, China) and NAC was diluted in nuclease-free water.

### 2.2. Protein Extraction and Western Blot Analysis

Total proteins of cells were extracted with lysis buffer (150 mM NaCl, 50 mM Tris-HCl, 1% Triton-X-100, 1 mM EDTA), EDTA-free PhosStop, and complete protease inhibitor (Roche Applied Science, Indianapolis, IN, USA). The protein concentration was determined by the bicinchoninic acid (BCA) protein assay kit (Sangon Biotech, Shanghai, China). Protein samples (15–20 μg) were run on polyacrylamide gels and transferred to PVDF membranes using TurboBlot (Bio-Rad, Hercules, CA, USA). Then, the blocked membrane was incubated with primary antibodies Nrf2 (Abcam, Cambridge, UK, 1:1000), BRD4 (Bethyl, Los Angeles, CA, USA, 1:500), KEAP1 (CST, Boston, MA, USA, 1:500), G6PD (Abcam, Cambridge, UK, 1:1000), H3 (CST, Boston, MA, USA, 1:1000), Tubulin (CST, Boston, MA, USA, 1:1000), and β-actin (Transgen, Beijing, China 1:1000) overnight and HRP-conjugated secondary antibody, Rabbit IgG (CST, Boston, MA, USA, 1:3000), and mouse IgG (CST, Boston, MA, USA, 1:3000) for 2 h. Signals were visualized using an ECL chemiluminescence detection kit (NCM, Suzhou, China) as per the manufacturer’s protocol on a Tanon 5200 Chemiluminescence image analyzer (Shanghai, China).

### 2.3. Chromatin Immunoprecipitation and PCR

Briefly, cells were crosslinked for 10 min with 1% (*v*/*v*) formaldehyde. Crosslinking was terminated for 5 min by adding a 1/10 volume of 1.25 M glycine and sonicated lysate to shear DNA into 200–500 bp fragments. Then, the chromatin was incubated with various antibodies (Nrf2, Abcam, ab62352; BRD4, CST, #13440; G6PD, Abcam, ab133525; IgG, and CST). Finally, the immunoprecipitated DNA was purified and amplified with gene-specific primers by quantitative PCR (qPCR). The primer sequences used for ChIP-qPCR were as follows: G6PD_Prom_F, 5′-ACGAGCAAACAGGCATATGA-3′ and G6PD_Prom_R, 5′-CCAAACTTGACTGCGCTCTAT-3′; KEAP1_Prom_F, 5′-GAAAGG AGCGGCGATTCTC-3′ and KEAP1_Prom_R, 5′-TGGAAGGGACAGTGAGAAGG-3′.

### 2.4. Co-Immunoprecipitation (Co-IP)

For co-immunoprecipitation (Co-IP) experiments, cells were lysed using IP lysis buffer (20 mM Tris-HCl pH 8, 137 mM NaCl, 1% nonidet-P-40 (NP-40)) and EDTA-free PhosStop and complete protease inhibitor (Roche Applied Science) on ice for 30 min. First, the protein was quantified after supernatant collection by centrifugation (1300 rpm, 15 min). Next, 500 µg protein extracts were mixed with 2.5 µL antibodies (Nrf2; BRD4) and incubated by rocking at 4 °C. After overnight incubation, 50 μL of magnetic beads (Thermo Scientific, Rockford, IL, USA) were added and the mixture continued to rock for 4 h. Finally, the beads were harvested and re-suspended in 20 μL of 2× sample buffer, followed by SDS-PAGE and immunoblotting

### 2.5. RNA Isolation and Quantitative RT-PCR Analysis

Total RNA was isolated from SCLC cells using TRIzol reagent (Thermo Scientific, Rockford, IL, USA) and reverse-transcribed using an Evo M-MLV Kit (Accurate Biology, Hunan, China). In addition, quantitative PCR was performed using an SYBR Green Premix Pro Taq HS qPCR Kit (Accurate Biology, Hunan, China) on a LightCycler 96^Ⓡ^ Instrument (Roche, Indianapolis, IN, USA). The primer sequences are as follow:

BRD4-F: 5′-ACCTCCAACCCTAACAAGCC-3′;
BRD4-R: 5′-TTTCCATAGTGTCTTGAGCACC-3′;
β-actin-F: 5′-TGTATGCCTCTGGTCGTACC-3′;
β-actin–R: 5′-CAGGTCCAGACGCAGGATG-3′;
G6PD-F: 5′-CCGGAAACGGTCGTACACTT-3′;
G6PD-R: 5′-ATGACGCTGTCTGCGCTT-3′;
KEAP1-F: 5′-TGGCCAAGCAAGAGGAGTTC-3′;
KEAP1-R: 5′-GGCTGATGAGGGTCACCAGTT-3′;
NQO1-F: 5′-CCTGCCATTCTGAAAGGCTGGT-3′;
NQO1-R: 5′- GTGGTGTGTGGAAAGCACTGCCT-3′;
FTL-F: 5′-CACCTACCTCTCTGGGCT-3′;
FTL-R: 5′-CAATTCGCGGAAGAAGTGGC-3′;
FTH1-F: 5′-CCAGAACTACCACCAGGACTC-3′;
FTH1-R: 5′-GTAAGTAGCTGGGCAGAGGCAA-3′.

And the gene ID accession numbers are as follow: BRD4 (23476), β-actin (60), G6PD (2539), KEAP1 (9817), NQO1 (1728), FTL (2512) and FTH1 (2495).

### 2.6. Cell Viability Assay

Cells (3000 per well) were seeded into 96-well plates and cultured for 24 h. The cells were then treated with different concentrations of drugs for 72 h. Cell viability was determined by the CellTiter-Glo Luminescent assay and luminescence was recorded using an Envision PerkinElmer porous plate microplate reader. The values were normalized, and the IC50 was calculated using GraphPad Prism (GraphPad Prism Software, San Diego, CA, USA).

### 2.7. Preparation of Cytoplasmic and Nuclear Extracts Assay

A total of 1 × 10^6^ cells were collected by centrifugation (1000 rpm, 5 min). After washing with cold PBS, the cells were incubated with 400 μL precooled buffer solution A (10 mM HEPES pH 7.9, 10 mM KCl, 0.1 mM EDTA, 1 mM DTT, protease inhibitor) and extracted for 30 min by stirring very gently. Then, 25 µL of 10% NP-40 was added to the mixture and allowed to be vortexed for 10 s. Then, the samples were centrifuged in a low-speed centrifuge (2000 rpm, 15 min, 4 °C). The resulting supernatant was centrifuged at high speed (10,000× *g*, 5 min, 4 °C), and collected the supernatant as the cytoplasmic fraction. Then, after washing the remaining precipitate, 50 µL cold buffer C (20 mM HEPES pH 7.9, 0.4 M NaCl, 1 mM EDTA, 1 mM DTT, protease inhibitor) was added and incubated on ice for 15 min. The supernatant was collected as nuclear extracts. Finally, samples were analyzed using immunoblot analysis.

### 2.8. Transcription Factor Prediction

Transcription factors were predicted using the AnimalTFDB 3.0 database, a comprehensive resource for annotation and prediction of animal transcription factors. The KEAP1 promoter was defined as 2000 bp upstream and 100 bp downstream from the KEAP1 transcription start site. The cut-off of the q-value was defined as 0.01 and transcription factors were included only if the predicted binding site was located in a positive strand.

### 2.9. Multi-Omics Data Analysis

The RNA-seq data for 17 different cancer types and associated clinicopathological information were downloaded from the TCGA database (TCGA. Available online: https://portal.gdc.cancer.gov/ (accessed on 16 November 2021)) [26]. Receiver operating characteristic (ROC) curves and prognostic analyses were performed using GraphPrism after the expression data of KEAP1 for specific cancer and its clinicopathological information were retrieved. Differential expression analysis across human cancers was carried out using a standard processing pipeline from the TIMER database (TIMER. Available online: https://cistrome.shinyapps.io/timer (accessed on 15 November 2021)), an interactive web resource for analyzing cancer OMICS data [27]. Finally, the comprehensive mutation profiles of KEAP1 were analyzed by a standard processing pipeline in the cBioPortal (cBioPortal. Available online: https://www.cbioportal.org (accessed on 12 January 2022)), a web-based database analyzing multidimensional cancer genomics data [28,29].

### 2.10. Analysis of Expression Data

Sequencing data (RNA-seq) from SCLC cell lines and general information for these cell lines were downloaded from https://portals.broadinstitute.org/ccle/data (accessed on 21 April 2021). In addition, transcriptome sequencing data from 81 human primary SCLC tumors and sample information were obtained from George et al., 2015. Sequencing data (RNA-seq) from 79 human primary SCLC tumors and microarray data from 18 SCLC and their matched normal tissues were downloaded from GSE60052 and GSE14956, respectively. Expression data for *KEAP1*, *G6PD*, and BET members were retrieved, analyzed, and displayed in scatter plots.

### 2.11. RNA Interference

For siRNA-mediated BRD4, cells were seeded into 6-well plates at 60% density and transfected with BRD4 siRNA or NC-siRNA using Effectene Transfection Reagent (QIAGEN, Dusseldorf, Germany) according to the manufacturer’s protocol. After 48 h of incubation, cells were harvested for western blot and RT-qPCR analysis. The siRNA sequences are shown:

siControl: 5′-UUCUCCGAACGUGUCACGUTT-3′,

       5′-ACGUGACACGUUCGGAGAATT-3′;

siBRD4#1: 5′-GCCAAATGTCTACACAGTATA-3′;

siBRD4#2: 5′-CAGTGACAGTTCGACTGATGA-3′.

### 2.12. SCLC Xenograft Mouse Models

Six-week-old athymic nude mice were subcutaneously injected with 5 × 10^6^ H82 cells in 100 mL of PBS and 100 mL of Matrigel (BD Biosciences, Franklin, NJ, USA). Drug treatment was initiated once the tumors reached 100 mm^3^. Before administration, JQ1 and RRx-001 were dissolved in DMSO and then injected into the tail vein at 40 mg/kg and 6 mg/kg, respectively, at a frequency of 4 injections every 5 days. The tumor size was measured with a caliper, and the following formula determined the tumor volume: tumor volume [mm^3^] = (tumor length × tumor width^2^)/2.

## 3. Results

### 3.1. KEAP1 Is UpRegulated and Correlated with Prognosis in Various Cancer Types

**Figure 1:**
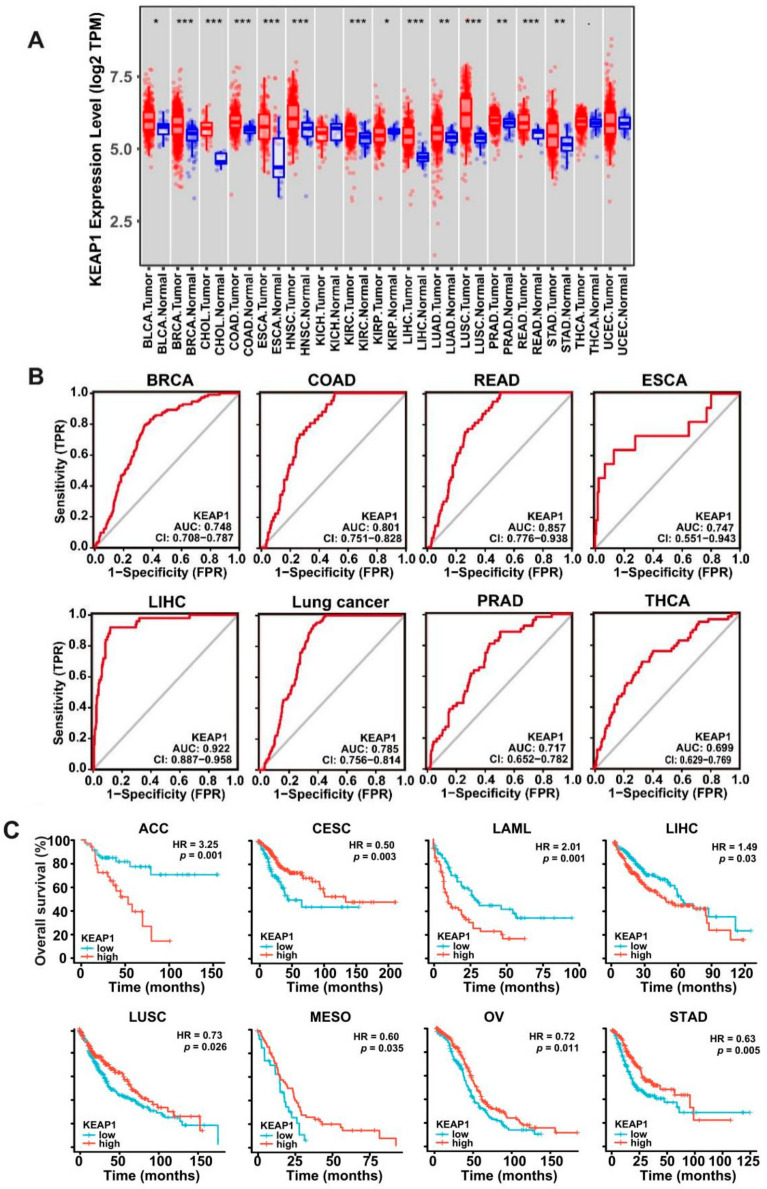
*KEAP1* expression across cancers and its prognostic implications. (**A**) Box plots showing the distribution of *KEAP1* mRNA expression in 17 types of cancer versus normal tissues based on the TCGA database. * *p* < 0.05, ** *p* < 0.01, *** *p* < 0.001. (**B**) ROC analysis of *KEAP1* expression for distinguishing cancers from normal tissues. BRCA, breast invasive carcinoma; COAD, colon adenocarcinoma; RSAD, rectum adenocarcinoma; ESCA, esophageal carcinoma; LIHC, liver hepatocellular carcinoma; PRAD, prostate adenocarcinoma; and THCA, thyroid carcinoma. (**C**) Kaplan–Meier analysis showing that *KEAP1* expression was linked to overall survival in eight cancer types based on the TCGA database. BLCA, Bladder Urothelial Carcinoma; BRCA, Breast invasive carcinoma; CHOL, Cholangio carcinoma; COAD, Colon adenocarcinoma; ESCA, Esophageal carcinoma; HNSC, Head and Neck squamous cell carcinoma; KICH, Kidney, Chromophobe; KIRP, Kidney renal papillary cell carcinoma; LIHC, Liver hepatocellular carcinoma; LUAD, Lung adenocarcinoma; LUSC, Lung squamous cell carcinoma; PRAD, Prostate adenocarcinoma; READ, Rectum adenocarcinoma; STAD, Stomach adenocarcinoma; THCA, Thyroid carcinoma; UCEC, Uterine Corpus Endometrial Carcinoma; ACC, Adrenocortical carcinoma; CESC, Cervical squamous cell carcinoma; LAML, Acute Myeloid Leukemia; MESO, Mesothelioma; OV, Ovarian serous cystadenocarcinoma.

To investigate the differential expression of *KEAP1* across cancers, a pan-cancer analysis of *KEAP1* expression was performed using the TIMER portal, an interactive tool for analyzing The Cancer Genome Atlas (TCGA) RNA-sequencing data. Bioinformatics analysis of 17 different types of cancer diseases revealed that *KEAP1* expression was significantly elevated compared with normal tissues in all primary tumors as compared with normal tissues except kidney chromophobe (KICH), thyroid carcinoma (THCA), and uterine corpus endometrial carcinoma (UCEC) (Figure 1A), indicating frequent elevated expression of *KEAP1* in cancer. To evaluate the diagnostic value of *KEAP1*, receiver operating characteristic curve (ROC) analysis was applied to determine the diagnostic efficiency of *KEAP1* expression in discriminating cancer patients from healthy individuals. The data showed that the area under the ROC curve (AUC) of *KEAP1* was larger than 0.7 in patients with invasive breast carcinoma (BRCA), colon adenocarcinoma (COAD), rectum adenocarcinoma (READ), esophageal carcinoma (ESCA), liver hepatocellular carcinoma (LIHC), lung cancer, prostate adenocarcinoma (PRAD), or thyroid carcinoma (THCA). Among them, the sensitivity and specificity of *KEAP1* in LIHC prediction were 0.920 and 0.874, respectively, under the optimal *KEAP1* expression cut-off value (Figure 1B, Supplementary Table S1). These data indicated that *KEAP1* possessed a conspicuous prognostic value in clinical practice. To investigate whether KEAP1 could serve as a prognostic marker for patient survival, overall survival (OS) curves were plotted by the Kaplan-Meier method according to the *KEAP1* expression level. The results demonstrated that *KEAP1* expression was significantly associated with overall survival. Patients with cervical squamous cell carcinoma and endocervical adenocarcinoma (CESC), lung squamous cell carcinoma (LUSC), mesothelioma (MESO), ovarian serous cystadenocarcinoma (OV), and stomach adenocarcinoma (STAD) with high *KEAP1* expression and patients with adrenocortical carcinoma (ACC), acute myeloid leukemia (LAML), and LIHC with low levels of *KEAP1* expression were predicted to have a high overall survival (Figure 1C). These results suggest that KEAP1 is aberrantly expressed in cancers and might be used as a potential biomarker to predict the prognosis of patients in a subset of cancers.

### 3.2. BRD4 Targets the KEAP1 Promoter and Regulates KEAP1 Expression in Lung Cancer

**Figure 2:**
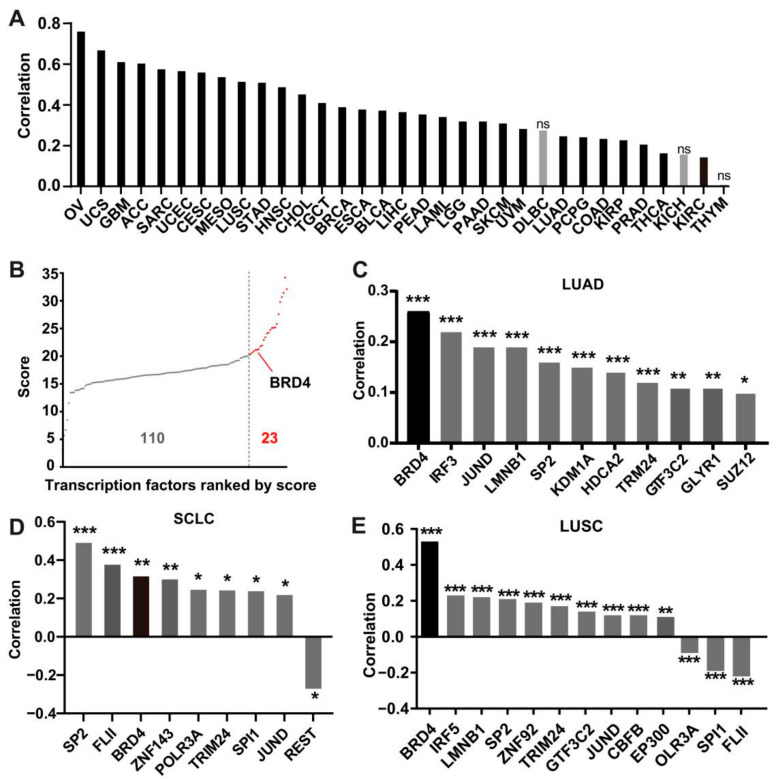
Bioinformatics analysis of factors modulating *KEAP1* expression. (**A**) Copy number variation (CNV) contribution to *KEAP1* expression using the MEXPRESS database. (**B**) Distribution of the predicted score sequencing of 139 transcription factors binding to the *KEAP1* promoter. (**C**–**E**) Histogram showing the mRNA expression correlation between *BRD4* and *KEAP1* based on the TCGA dataset-LUAD (**C**), TCGA dataset-LUSC (**D**), and in SCLC primary tumors (E, *n* = 81). ns > 0.05; * *p* < 0.05; ** *p* < 0.01; *** *p* < 0.001 (Student’s *t*-test). SCLC, small cell lung cancer; UCS, Uterine Carcinosarcoma; GBM, Glioblastoma multiforme; SARC, Sarcoma; TGCT, Testicular Germ Cell Tumors; LGG, Brain Lower Grade Glioma; PAAD, Pancreatic adenocarcinoma; SKCM, Skin Cutaneous Melanoma; UVM, Uveal Melanoma; DLBC, Lymphoid Neoplasm Diffuse Large B-cell Lymphoma; PCPG, Pheochromocytoma and Paraganglioma; KIRC, Kidney renal clear cell carcinoma; and THYM, Thymoma.

To explore the underlying mechanisms leading to the dysregulation of KEAP1 in cancer, we first characterized genomic alterations of the *KEAP1* gene. Mutations, copy number changes, and structural variants for *KEAP1* were analyzed across human cancers using the cBioPortal database. The results showed that high-level copy number gains of KEAP1 were observed in uterine carcinosarcoma, ovarian serous cystadenocarcinoma, and sarcoma. In contrast, we only observed relatively low-level copy number gains and losses of KEAP1, even though a high mutation rate of KEAP1 was detected in lung adenocarcinoma (LUAD) and squamous cell carcinoma (Supplementary Figure S1A). Given that somatic copy number alterations affect gene expression, we integrated copy number variation (CNV) and RNA-seq data from the TCGA database to determine the association of CNV and *KEAP1* expression. As shown in Figure 2A, the mRNA level of *KEAP1* was significantly positively correlated with the changes in somatic copy number in most cancer types examined except DLBC, KICH, and THYM (Figure 2A). Although LUAD and LUSC have significant correlation coefficients, OV exhibits the highest correlation coefficient across cancers. Since the expression of genes of interest could be regulated at the transcriptional level, we then sought to explore the transcription factors responsible for the up-regulation of *KEAP1*. We focused on lung cancer since relatively low-level copy number changes were found in lung cancer. We first predicted the potential transcription factor binding sites at the promoter of *KEAP1* using the HumanTFDB 3.0 database. A total of 133 transcription factors showed the possibility of binding to the *KEAP1* promoter. To narrow down potential transcription factors, we chose transcription factors whose predicted scores were larger than 20, and finally, 23 genes were selected for further analysis (Figure 2B). Pearson correlation analysis was performed to assess the correlation between the 23 potential transcription factors and *KEAP1* expression in lung cancer. Interestingly, BRD4 showed a strong positive correlation with *KEAP1* expression in three major lung cancer subtypes, including LUAD, LUSC, and SCLC (Figure 2C–E). We also retrieved ChIP-Seq data from the UCSC database and found that BRD4 was enriched as the promoter of KEAP1 in H2171 and PC9 cells and, to a lesser extent, in HCT-116 cells (Supplementary Figure S1B). These data suggest that BRD4, as an upstream regulator of *KEAP1*, might regulate gene expression by binding to the KEAP1 promoter in cancer cells.

### 3.3. The Positive Association between KEAP1 and BRD4 in SCLC

Lung cancer comprises two major histological types: non-small-cell lung cancer (NSCLC) and small-cell lung cancer (SCLC). Compared with non-small cell lung cancer, *KEAP1* and *BRD4* expression were much higher in SCLC cells than in NSCLC cells based on publicly available RNA-seq data from Cancer Cell Line Encyclopedia (CCLE) and microarray data from Genomics of Drug Sensitivity in Cancer (GDSC) (Supplementary Figure S2A,B). Therefore, we decided to explore the potential transcriptional regulation of *KEAP1* by BRD4 in SCLC cells by first evaluating multiple SCLC microarray and RNA-seq data sets for *KEAP1* and *BRD4* expression. Analysis of the GSE149507 data set (18 paired SCLC and adjacent normal tissues) showed that both KEAP1 and BRD4 were elevated compared with adjacent normal tissues (Supplementary Figure S2C). Similar results were also observed in the GSE60052 data set (data not shown). Interestingly, survival analysis using the Kaplan-Meyer method showed that high expression of *KEAP1* and *BRD4* was associated with better overall and progression-free survival in SCLC. These results indicate that both *KEAP1* and *BRD4* are highly expressed and that high *BRD4* and *KEAP1* expression consistently contributes to a better prognosis of SCLC patients (Supplementary Figure S2D,E).

**Figure 3:**
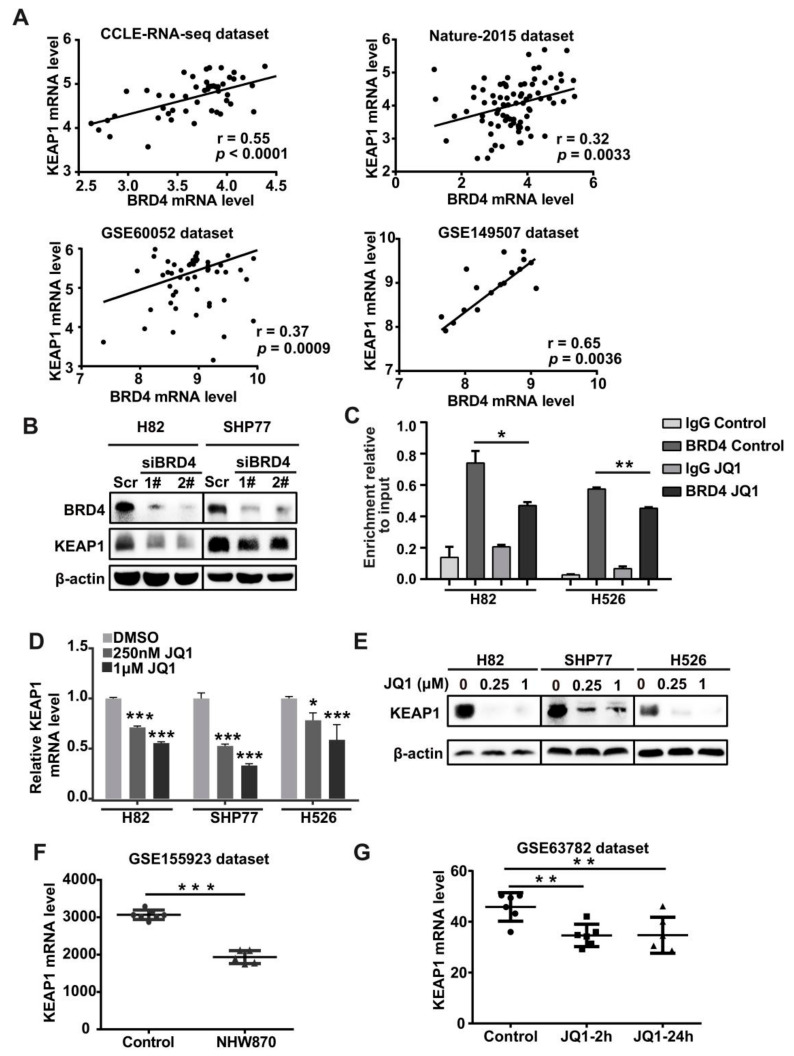
BRD4 targets the *KEAP1* promoter and dominantly regulates *KEAP1* expression. (**A**) Scatter plot showing the correlation between *KEAP1* and *BRD4* RNA expression that was demonstrated based on the four databases. (**B**) Western blot analysis of BRD4 and KEAP1 upon knockdown of *BRD4* in SCLC cells. (**C**) ChIP-qRT-PCR experiment indicating the direct binding of BRD4 to the KEAP1 promoter in H82 and H526 cells after 48-h exposure to JQ1 or 0.01% DMSO (vehicle). The effect of JQ1 on the DNA-binding efficiency of BRD4 to the *KEAP1* promoter was observed. (**D**,**E**) The effect of JQ1 on the *KEAP1* expression by RT-qPCR (**D**) or western blot (**E**) in H82, SHP77, and H526 cells. (**F**,**G**) Scatter plots showing *KEAP1* expression on BET inhibitor NHW870 (**F**) or JQ1 (**G**) based on the GSE155923 and GSE63782 datasets. * *p* < 0.05; ** *p* < 0.01; *** *p* < 0.001 (Student’s *t*-test).

To further characterize the correlation between *KEAP1* and *BRD4* at the mRNA level in SCLC, we interrogated multiple SCLC RNA-seq and microarray data sets for *KEAP1* and *BRD4* expression. A primary SCLC data set generated by George et al. [30] (81 human primary SCLC samples) displayed that *BRD4* expression was highly positively correlated with *KEAP1* expression. Similar correlation patterns were observed in the GSE149507, GSE60052, and CCLE SCLC RNA-seq data sets (Figure 3A). In contrast, *BRD2* and *BRD3*, two additional BET (bromodomain and extra-terminal domain) family members, exhibited no or low correlation coefficients with *KEAP1*. These data suggest that BRD4 might be the primary BET member responsible for the regulation of *KEAP1*. To provide mechanistic evidence of the potential regulation of *KEAP1* by *BRD4*, we silenced *BRD4* by siRNA in H82 and SHP77 SCLC cells, and then *BRD4* and *KEAP1* expression was validated by RT-qPCR and Western blotting. As depicted in Figure S3B, effective knockdown of BRD4 markedly decreased *KEAP1* expression in both H82 and SHP77 cells (Supplementary Figure S3A,B). Following the RT-qPCR results, western blot analysis demonstrated a down-regulation of KEAP1 upon *BRD4* knockdown at the protein level (Figure 3B, Supplementary Figure S5A). Then, we performed BRD4 chromatin immunoprecipitation (ChIP) followed by qPCR analysis to evaluate whether BRD4 transcriptionally activated *KEAP1*. The results showed that BRD4 is directly bound to the promoter region of *KEAP1*. As expected, treatment with JQ1, a specific inhibitor of BRD4, led to markedly decreased binding of BRD4 at the promoter of KEAP1 in H82 and H526 cells (Figure 3C). Notably, JQ1 treatment resulted in a dose-dependent down-regulation of KEAP1 at the mRNA and protein levels in H82, SHP77, and H526 cells (Figure 3D,E, Supplementary Figure S5B). We also extracted *KEAP1* expression data from two GEO datasets and analyzed the effect of BET inhibitors on the *KEAP1* expression. The results showed that JQ1 caused a dose-dependent decrease in *KEAP1* in four SCLC cell lines in the GSE63782 data set. We also found that NHW870, another BET inhibitor, down-regulated KEAP1 in an SCLC PDX model (Figure 3F,G). Altogether, these data validated that BRD4 directly binds to the *KEAP1* promoter and positively regulates its expression in SCLC cells.

### 3.4. BETi and Nrf2i Synergistically Inhibit SCLC Cell Proliferation

**Figure 4:**
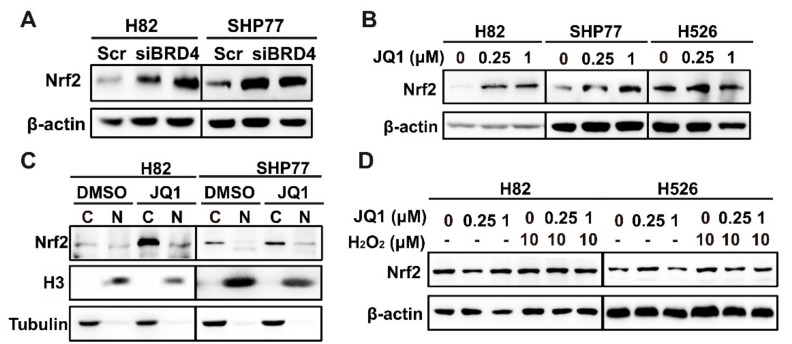

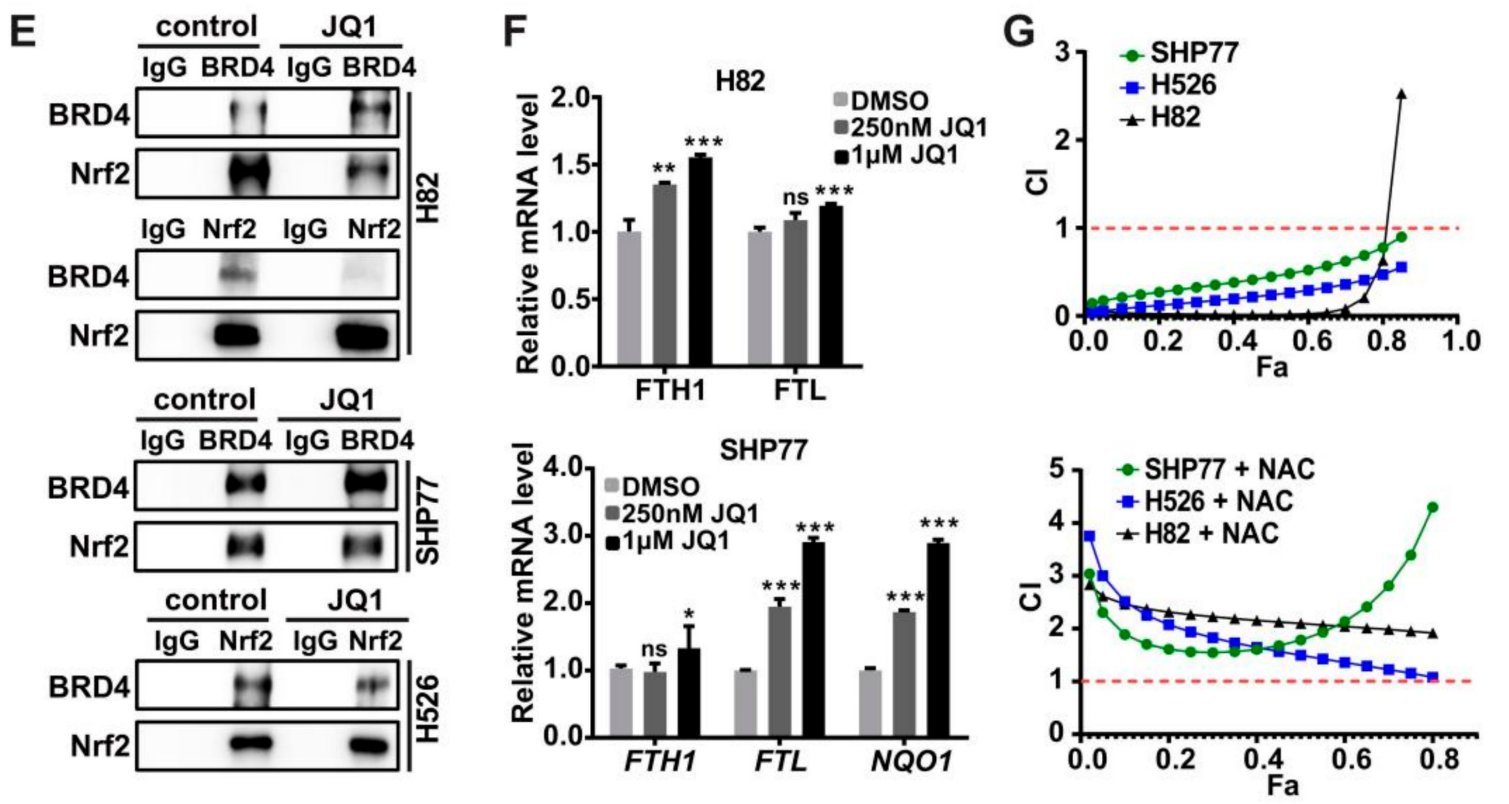
Blocking BRD4 results in the accumulation of Nrf2. (**A**,**B**) Western blot analysis of Nrf2 upon BRD4 knockdown or JQ1 treatment (**B**) in H82 and SHP77 cells. (**C**) Western blot analysis indicating the distribution of Nrf2 upon JQ1 treatment in H82 and SHP77 cells Tubulin serves as a reference protein in the cytoplasm and H3 as a reference protein in the nucleus. (**D**) Western blot analysis of Nrf2 in H82 and H526 cells with or without H_2_O_2_ after treatment with JQ1. (**E**) Co-IP experiments demonstrating the interaction of BRD4 and Nrf2 upon JQ1 treatment. (**F**) RT-qPCR analysis of *FTH1, FTL,* and *NQO1* upon JQ1 treatment in H82 and SHP77 cells. (**G**) CellTiter-Glo Luminescent assays demonstrating the effects of the combination of ATRA and JQ1 with (lower panel) or without NAC (upper panel) in H82, SHP77, and H526 cells. ns > 0.05; * *p* < 0.05; ** *p* < 0.01; *** *p* < 0.001 (Student’s *t*-test).

The KEAP1-Nrf2 axis is one of the most important cellular defense pathways against oxidative and electrophilic stress. We analyzed several public data sets for nuclear factor (erythroid-derived 2)-like 2 (NFE2L2) expression. As shown in Figure S3C, NFE2L2 was lower in SCLC tissues/cells than adjacent non-tumor tissues in the GSE145907 data set and LUAD cells in the CCLE RNA-seq data set (Supplementary Figure S3C). Impaired *KEAP1* expression mediated by BRD4 might influence its association with Nrf2, promoting Nrf2 stability by preventing Nrf2 degradation. Therefore, we evaluated *Nrf2* expression by RT-qPCR and Western blotting to determine whether *Nrf2* expression was affected with BRD4 knockdown or JQ1 treatment. Notably, knockdown of BRD4 using siRNA or treatment of H82 and H526 cells with JQ1 had no significant effect on the transcription of NFE2L2 (Supplementary Figure S3D,E). However, BRD4 depletion triggered the accumulation of Nrf2 at the protein level in both H82 and SHP77 cells. Similarly, JQ1 treatment induced a dose-dependent up-regulation of NFR2 in H82, SHP77, and H526 cells (Figure 4A,B, Supplementary Figure S5C,D). Following the down-regulation of KEAP1, Nrf2 would no longer be sequestered by KEAP1 and translocate and accumulate in the nucleus. We then checked whether the nuclear fraction of Nrf2 increased following JQ1 treatment. The nuclear-cytoplasmic separation assay demonstrated that Nrf2 was more translocated and retained in the nucleus in the presence of JQ1 in H82 and SHP77 cells (Figure 4C, Supplementary Figure S5E). External oxidative stress also disassociates KEAP1 from Nrf2 and leads to the accumulation of Nrf2 so we treated cells with hydrogen peroxide in the presence or absence of JQ1 and detected Nrf2 protein by western blotting. As shown in Figure 4D, the perturbation of redox homeostasis by hydrogen peroxide could further lead to the dose-dependent accumulation of Nrf2 in H82 and H526 cells. These results suggested that either *BRD4* depletion or blocking the binding of BRD4 to the *KEAP1* promoter by JQ1 indeed activates the KEAP1-Nrf2 signaling pathway. In addition, a previous study reported that Fs(1) h, the fly ortholog of BET member, physically interacts with CncC, the ortholog of Nrf2 in Drosophila, and inhibits its activity at the posttranslational level. We tested the possible interaction between BRD4 and Nrf2 in SCLC cells by co-immunoprecipitation (Co-IP) analysis. The co-IP experiment unveiled an interaction between BRD4 and Nrf2 in H82, SHP77, and H526 cells. Importantly, JQ1 treatment weakened the binding rate of Nrf2 and BRD4 (Figure 4E). These data suggest that BRD4 can modulate Nrf2 activity through indirect regulation by KEAP1 and direct protein-protein regulation.

Furthermore, Nrf2 translocation from the cytoplasm to the nucleus will allow it to bind to antioxidant-response elements and activate a wide battery of genes. Therefore, we sought to determine the effect of JQ1 on Nrf2 targets. RT-qPCR analysis showed that JQ1 treatment significantly up-regulated the expression of antioxidant-related genes that are downstream of Nrf2 (NQO1, FTL, and FTH1) in H82 and SHP77 cells (Figure 4F). BRD4 has been implicated in a broad spectrum of human cancers and is increasingly appreciated as a promising anticancer target, including SCLC. Both intrinsic and acquired resistance to BET inhibitors have started to draw much attention and the underlying mechanisms leading to resistance in SCLC are still largely unexplored. Activating Nrf2 by *BRD4* silencing or BET inhibitors could protect SCLC cells from oxidative stress, therefore promoting cell survival. To test our hypothesis, we treated SCLC with JQ1 in combination with ATRA, a well-recognized inhibitor of Nrf2, and evaluated the potential synergistic inhibitory effect. We found that the combination of JQ1 and ATRA indeed showed strikingly synergistic anti-tumor activity in H82, SHP77, and H526 cells (Figure 4G). However, the addition of N-acetyl L-cysteine (NAC), a well-known reactive oxygen species (ROS) scavenger, prevented the synergistic effects induced by the combined treatment with JQ1 and ATRA in the cell lines examined (Figure 4G). These results strongly suggest that JQ1-mediated activation of Nrf2 facilitates cell survival and that ATRA may promote the cytotoxicity of JQ1 by interfering with Nrf2 and increasing ROS levels in SCLC cells.

### 3.5. Co-Targeting BRD4 and G6PD Suppresses SCLC In Vitro and In Vivo

**Figure 5:**
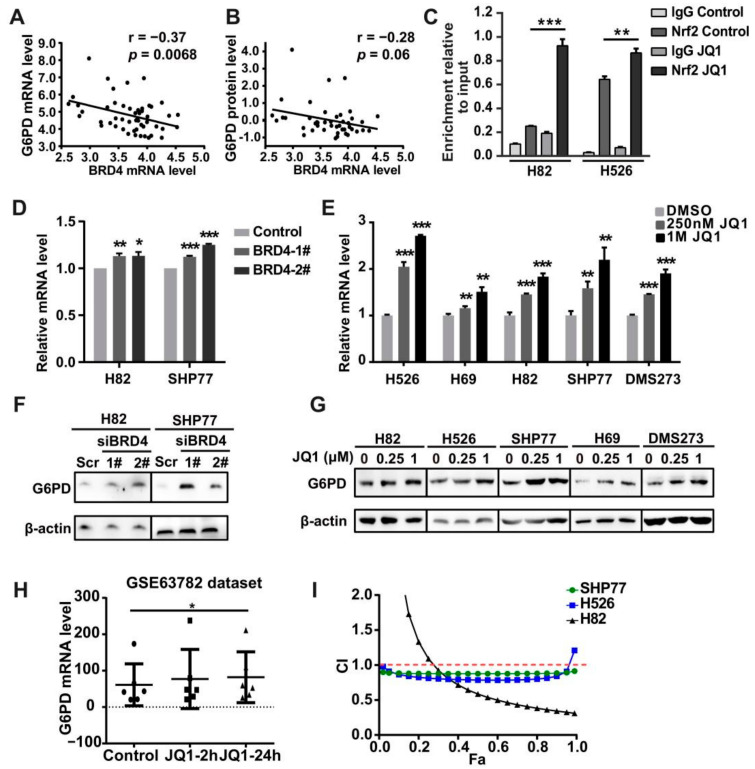
Nrf2 transcriptionally regulates *G6PD* in SCLC cells. (**A**,**B**) Scatter plots showing the correlation between *BRD4* expression and the expression of *G6PD* at mRNA (**A**) and protein (**B**) levels. (**C**) ChIP-qRT-PCR experiment indicating the direct binding of Nrf2 to the G6PD promoter in H82 and H526 cells after 48 h exposure to JQ1 or 0.01% DMSO (vehicle). (**D**,**E**) RT-qPCR analysis of *G6PD* upon *BRD4* silencing (**D**) in H82 and SHP77 cells and JQ1 treatment (**E**) in five SCLC cell lines. (**F**,**G**) Western blot analysis of G6PD upon BRD4 silencing (**F**) in H82 and SHP77 cells and JQ1 treatment (**G**) in five SCLC cell lines. (**H**) Scatter plots showing *G6PD* expression upon JQ1 treatment based on the GSE63782 dataset. (**I**) CellTiter-Glo Luminescent assays demonstrating the synergistic effects of the combination of RRx-001 and JQ1 in H82, SHP77, and H526 cells. * *p* < 0.05; ** *p* < 0.01; *** *p* < 0.001 (Student’s *t*-test).

Several studies have shown that Nrf2 up-regulates *G6PD* expression [31]. Therefore, we first interrogated a couple of data sets for *G6PD* expression in SCLC. Compared with that in LUAD cells, the expression of *G6PD* in SCLC cells was also remarkably lower in the CCLE RNA-seq data set. Moreover, *G6PD* expression was much lower in human primary SCLC tissues than in adjacent non-tumorous tissues in the GSE149507 data set (Supplementary Figure S4A). Notably, in SCLC cell lines, we found that G6PD expression at both the RNA and protein levels was significantly negatively correlated with *BRD4* expression but not with *BRD2* and *BRD3* expression (Figure 5A,B, Supplementary Figure S4C,E). Importantly, knockdown of BRD4 stimulated G6PD mRNA and protein expression in H82 and SHP77 cells (Figure 5D,F, Supplementary Figure S6A). Similarly, JQ1 treatment enhanced *G6PD* mRNA expression and protein expression in SCLC cells or tissue samples (Figure 5E,G, Supplementary Figure S6B). Consistently, treatment with JQ1 also caused a time-dependent up-regulation of G6PD in SCLC cells based on the GSE63782 dataset (Figure 5H). Although we observed the binding of BRD4 to the *G6PD* promoter by ChIP-PCR, treatment with JQ1 had a minimal effect on the change in the association of BRD4 with the promoter region of *G6PD* (data not shown). Therefore, we wondered whether Nrf2 rather than BRD4 directly binds to the promoter region of *G6PD* and activates its expression. The ChIP-qPCR analysis demonstrated that Nrf2 was enriched in the G6PD promoter in the basal state. Furthermore, the addition of JQ1 robustly increased the binding of Nrf2 at the promoter of *G6PD* in H82 and SHP77 cells (Figure 5C). These results indicated that Nrf2, rather than BRD4, directly binds to the *G6PD* promoter and controls its expression in SCLC cells.

RRx-001 is an effective inhibitor of G6PD and has been used in several clinical trials for SCLC, including a phase II clinical trial in which sensitization was performed in SCLC patients with acquired resistance to first-line chemotherapy with the etoposide plus cisplatin (EP) regimen [27,28]. To explore whether RRx-001 could enhance the cytotoxicity of JQ1 in SCLC cells, we incubated SCLC cells with RRx-001 and JQ1 at different concentrations and measured cell viability after 72 h. The experimental results showed that the combined use of RRx-001 and JQ1 generated a strong synergistic effect on cell viability in H82, SHP77, and H526 cells (Figure 5I).

**Figure 6:**
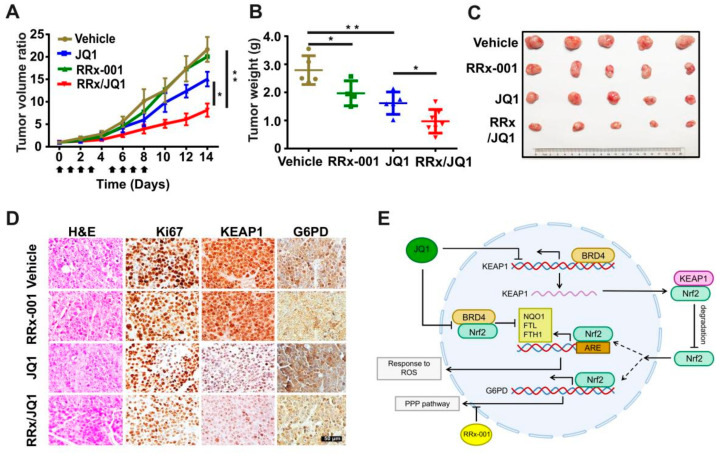
Co-targeting BRD4 and G6PD suppresses SCLC in vivo. (**A**) Tumor volume curves of H82 xenograft mice treated with RRx-001, JQ1, or a combination of RRx-001 and JQ1. The arrow represents the time of drug injection. (**B**) Tumor weights of H82 xenograft mice after 14 days of drug treatment. (**C**) Imaging of representative tumors excised at each group’s end of the experiment. (**D**) Representative immunohistochemistry images of Ki67, KEAP1, and G6PD on each group. Scale bar, 50 μm. (**E**) Model for targeting the BRD4-KEAP1-Nrf2-G6PD axis in SCLC cells. RRx, RRx-001. * *p* < 0.05; ** *p* < 0.01 (Student’s *t*-test).

Furthermore, to test the effect of the combination strategy in vivo, subcutaneously xenografted SCLC in nude mice was established. We then treated these mice with PBS, JQ1, RRx-001, or a combination of JQ1 with RRx-001 for ten days. Indeed, the outgrowth of the tumors in the combination treatment group was significantly slower than that in the RRx-001 or JQ1 only treatment group (Figure 6A). This synergistic effect of the combination strategy on tumorigenicity was also confirmed by measurements of the total weight or size of subcutaneous tumors excised from mice 14 days post-injection (Figure 6B,C). Notably, no animal death occurred during the 2 weeks of drug treatment and the weight of treated mice remained similar to that of control mice (Supplementary Figure S4G), suggesting that the combination of RRx-001 and JQ1 is well tolerated. Simultaneously, IHC staining of mouse tumors showed that combination treatment significantly reduced the expression levels of Ki67 and KEAP1 in subcutaneous tumor tissue (Figure 6D). In accordance with our observation, *G6PD* expression was also increased upon NHW870 treatment in the small cell lung cancer PDX LX-95 model (Supplementary Figure S4F). These in vivo experimental results confirmed the JQ1/RRx-001-mediated synergistic effect observed in vitro and indicated that JQ1-induced G6PD up-regulation might influence the therapeutic efficacy of BET inhibitors.

## 4. Discussion

The KEAP1-Nrf2 axis is the master regulator of cellular and organismal defense against oxidative and electrophilic assaults. The PPP pathway represents the principal cellular source of NADPH, the major cellular reductant [5]. In theory, the KEAP1-Nrf2 and PPP pathways might coordinate the regulation of redox homeostasis. However, currently, there are minimal data on the crosstalk between these two signaling pathways. In this study, the TCGA database data mining identified that KEAP1 is up-regulated in most cancer types. In addition, prognostic analysis based on TCGA expression data demonstrated that the *KEAP1* expression level exhibits potential as a prognostic marker in several cancers. Integrative analysis of different datasets unveiled that KEAP1 expression might be regulated at the genomic and transcriptional levels. Furthermore, we found that BRD4 expression was positively correlated with *KEAP1* expression and was predicted to be a factor binding to the *KEAP1* promoter in lung cancer. Using SCLC as a model, we demonstrated that *BRD4* associates with the promoter region of *KEAP1* and activates its expression. The accumulation of Nrf2 in the nucleus by KEAP1 down-regulation or inhibition by JQ1 and a decrease in the BRD4-Nrf2 interaction, led to the enrichment of Nrf2 in the *G6PD* promoter, inducing G6PD activation in SCLC. Our investigations identify that *BRD4* may regulate the activity of Nrf2 in both a KEAP1-dependent and -independent manner, providing novel insight into the regulatory mechanisms of redox homeostasis in SCLC.

Our investigations emphasize the critical roles of the KEAP1-Nrf2 system in cancers. Higher *KEAP1* expression is correlated with *BRD4* expression and higher expressions of both genes are associated with better overall survival in multiple cancer types, suggesting that tight control of the KEAP1-Nrf2 pathway might be necessary for maintaining redox homeostasis in cancer cells and cancer cell survival. Although our study only focused on SCLC, the molecular regulation of KEAP1-Nrf2-G6PD by BRD4 might be beyond SCLC. Future investigation of this regulatory network in other cancer types might be warranted.

Two significant findings can be summarized as follows (Figure 6E): (I) BRD4 regulates Nrf2 and its downstream G6PD in a KEAP1-dependent or KEAP1-independent manner. In the former, BRD4 can directly bind to the promoter of KEAP1 as a transcription factor to promote the transcription of KEAP1. Up-regulation of KEAP1 facilitates Nrf2 degradation and blocks the binding of Nrf2 to the G6PD promoter, ultimately leading to the down-regulation of the PPP pathway. In the latter, BRD4 directly binds to Nrf2 through acetylated lysine residues and suppresses Nrf2 activity, ultimately impairing the cell’s ability to respond to oxidative stress. (II) Metabolic remodeling of Nrf2 in response to oxidative stress is one of the mechanisms contributing to JQ1 resistance in SCLC, but this resistance can be solved by combining RRx-001 and JQ1.

BETis are currently being actively evaluated in clinical trials for several cancer types. Concern regarding resistance to BETis has been raised and some researchers have started to address this issue since resistance to BETis will limit their clinical efficacy. A number of reports have illustrated that activation of the PI3K/AKT pathway [19], or AKT–mTORC1 [32], is one of the mechanisms of acquired JQ1 resistance. For example, studies have demonstrated that BETi resistance is mediated by adaptive kinome reprogramming in ovarian cancer (OC) [33], neuroblastoma [19], or SPOP-mutated prostate cancer [32]. However, the underlying mechanisms responsible for intrinsic resistance to BET inhibition remain undetermined. Based on our results, we propose that activation of Nrf2-G6PD might be a molecular mechanism underlying the intrinsic resistance to BETis. Therefore, we proposed a new mechanism for intrinsic resistance to BETis and the enhancement of metabolic reprogramming and oxidative stress capacity of tumors in response to BET inhibition, thus providing a new direction for addressing resistance to BETis. Furthermore, our study also suggests the feasibility of a novel combination of JQ1/RRx-001 in the clinical treatment of patients with SCLC to overcome this type of resistance.

G6PD, the core rate-limiting enzyme in the PPP pathway, acts as a gatekeeper of PPP flux and plays a pivotal role in maintaining redox homeostasis. RRx-001 is a pleiotropic anticancer agent with activity mediated primarily through increased nitric oxide (NO) production and G6PD inhibition. In a phase II study called QUADRUPLE THREAT (NCT02489903), RRx-001 combined with platinum doublet chemotherapy was well tolerated. A phase III clinical study, REPLATINUM (NCT03699956), is also underway to evaluate RRx-001 as third- or further-line treatment for small-cell lung cancer after platinum-based chemotherapy and checkpoint inhibitor therapy [34]. Studies have also shown that RRx-001 followed by re-challenging with platinum plus etoposide chemotherapy is associated with promising results in clinical trials. Interestingly, RRx-001 has been shown to target colon cancer stem cells and reduce the expression levels of the Wnt pathway components and target genes, including c-Myc [35]. The authors suggest that RRx-001 should be used to treat c-Myc over-expressed tumors. Down-regulation of c-Myc by RRx-001 might also contribute to the synergistic activity by RRx-001-JQ1 in SCLC cells. In that case, RRx-001 not only acted as an inhibitor of G6PD but also inhibited c-Myc expression in combination with JQ1 in SCLC. Whether RRx-001 modulates c-Myc or c-Myc signaling in SCLC cells awaits further investigation.

In addition, we found a significant positive correlation between KEAP1 and BRD4 at the mRNA level in most cancer types. This correlation suggests that the regulatory mechanism of BRD4 on KEAP1 and G6PD may be beyond SCLC and may exist in a variety of cancers which provides a new basis for overcoming drug resistance to BETis in other cancer types. Moreover, we speculate that cancer cells with high BRD4 expression may depend more on the regulatory mechanism described here. Given that BRD4 is frequently elevated in cancer cells compared to normal cells, co-targeting of BRD4 and the KEAP1-Nrf2-G6PD axis might not only maximize the clinical efficacy of BET inhibitors but also cause fewer side effects in patients.

## 5. Conclusions

In conclusion, we identify a transcriptional regulatory mechanism for KEAP1 and G6PD and provide a strong rationale for therapies that include Nrf2i or G6PDi combined with BRD4 inhibitors for SCLC patients in the clinic.

## Data Availability

This study analyses the public data sets, the data can be found here: https://portal.gdc.cancer.gov/ (accessed on 16 November 2021), https://portals.broadinstitute.org/ccle/data (accessed on 21 April 2021), https://www.cbioportal.org (accessed on 12 January 2022) and https://cistrome.shinyapps.io/timer (accessed on 15 November 2021).

## Supplementary Materials

The following supporting information can be downloaded at: https://www.mdpi.com/article/10.3390/antiox11040661/s1, Figure S1: BRD4 is a potential regulator of *KEAP1* expression; Figure S2: Expression and clinical characteristics of BRD4 and KEAP1 in lung cancer; Figure S3: Knocking down BRD4 affects the KEAP1-Nrf2 axis; Figure S4: Correlation between BRD4 and G6PD; Figure S5: The quantification data of Figure 3 and Figure 4; Figure S6: The quantification data of Figure 5; Table S1: ROC analysis of KEAP1 expression for distinguishing cancers from normal tissues.
