# Supplementary material for: BRD4 Targets the KEAP1-Nrf2-G6PD Axis and Suppresses Redox Metabolism in Small Cell Lung Cancer
Source: Antioxidants (Basel). 2022 Mar 29;11(4):661. doi: 10.3390/antiox11040661 (PMC9029261; doi:10.3390/antiox11040661)
Supplement: Supplementary file 1 [file antioxidants-11-00661-s001.zip › antioxidants-1632119-supplementary.pdf]

antioxidants

Supplementary Materials: BRD4 Targets the KEAP1-Nrf2-G6PD Axis and Suppresses Redox Metabolism in Small Cell Lung Cancer

Yang Lv [1,2,3,†], Xiaotong Lv [1,2,3,†], Jiahui Zhang [1,2,3], Guozhen Cao [1,2,3], Changzhi Xu [4], Buchang Zhang [4] and Wenchu Lin [1,3,*]

[1] High Magnetic Field Laboratory, Hefei Institutes of Physical Science, Chinese Academy of Sciences, Hefei 230031, China; zwscjmx@mail.ustc.edu.cn (Y.L.); lvxt@mail.ustc.edu.cn (X.L.); zjhui@mail.ustc.edu.cn (J.Z.); cgzhwaxx@mail.ustc.edu.cn (G.C.)
[2] University of Science and Technology of China, Hefei 230026, China
[3] Key Laboratory of High Magnetic Field and Ion Beam Physical Biology, Hefei Institutes of Physical Science, Chinese Academy of Sciences, Hefei 230031, China
[4] Institutes of Physical Science and Information Technology, Anhui University, Hefei 230601, China; xcz@ahu.edu.cn (C.X.); zhbc@ahu.edu.cn (B.Z.)
* Correspondence: wenchu@hmfl.ac.cn; Tel.: +86-551-65593499
† These authors contributed equally to this work.

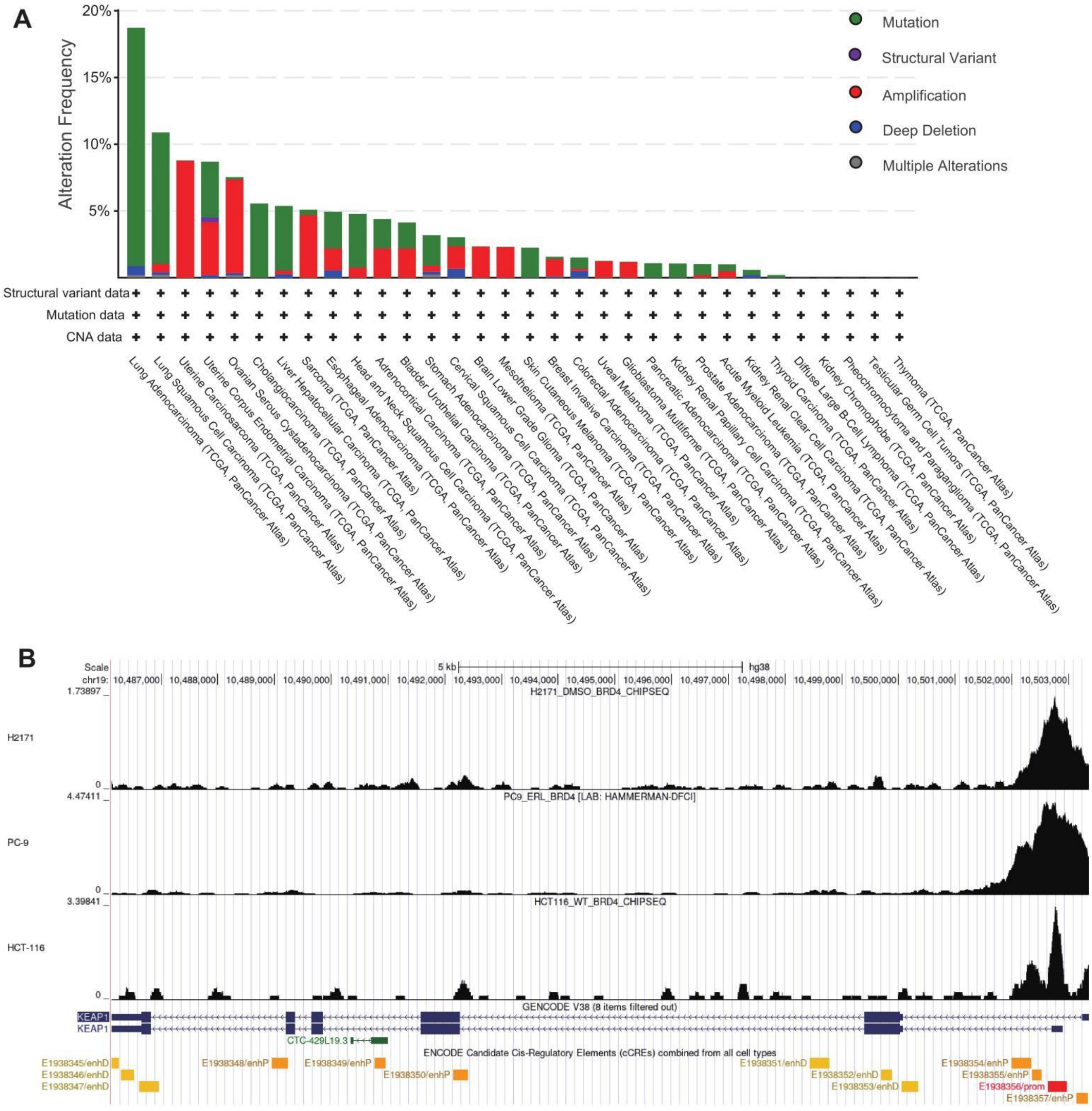

Supplementary Figure S1. BRD4 is a potential regulator of KEAP1 expression. A) Mutation frequency of KEAP1 in different cancer types in cBioPortal database. **B**) The peak diagram of BRD4 on KEAP1 genome in H2171, PC9 and HCT116 cells was obtained by UCSC genome browser.

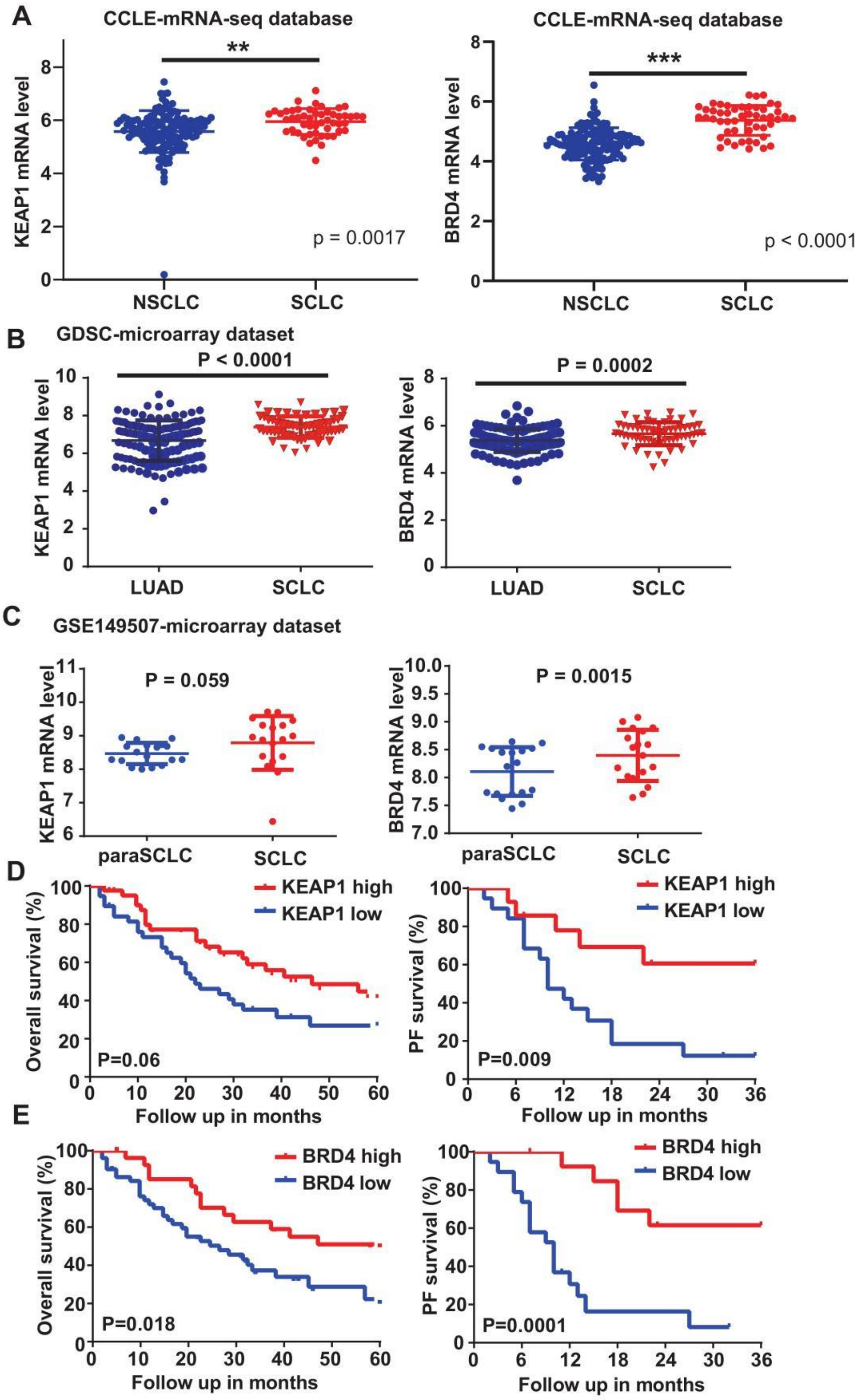

Supplementary Figure S2. Expression and clinical characteristics of BRD4 and KEAP1 in lung cancer. A) The mRNA expression of KEAP1 and BRD4 in NSCLC and SCLC cell lines was obtained from CCLE database. Blue: NSCLC; Red: SCLC. **B**) The mRNA expression levels of KEAP1 and BRD4 in LUAD and SCLC were obtained from GDSC database. Blue: LUAD; Red: SCLC. **C)** obtained KEAP1 and BRD4 expression levels in SCLC and para-carinoma tissues from GSE database.

D–E) The relationship between KEAP1 or BRD4 expression levels and OS or PF of SCLC was calculated according to nature-2015 database. *P < 0.05; **P < 0.01; ***p < 0.001 (Student's t test).

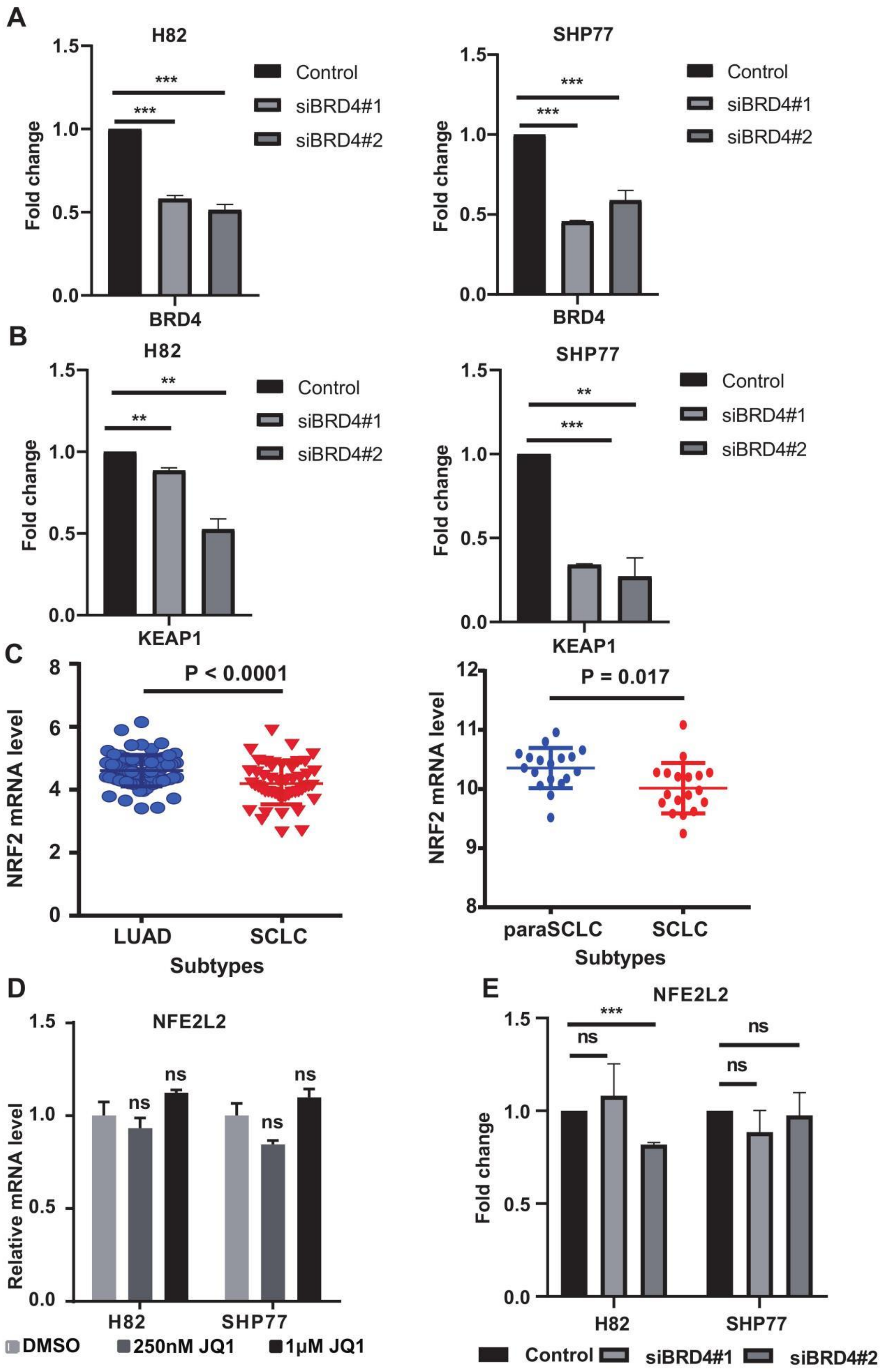

Supplementary Figure S3. Knocking down BRD4 affects the KEAP1-Nrf2 axis. A-B,E) The effect of knockdown BRD4 by using siBRD4#1 or siBRD4#2 on the BRD4 (**A**), KEAP1 (**B**) and NFE2L2 (**E**) expression by RT-qPCR in H82 and SHP77 cells. (**C**) mRNA expression of NFE2L2 in LUAD and SCLC cell lines was obtained from CCLE database (left), and the mRNA expression of NFE2L2 in paracancer and SCLC tissues was obtained from SE145907 database. (**D**) The effect of JQ1 treatment on NFE2L2 expression in H82 andSHP77 cells was detected by RT-qPCR. ns > 0.05, *P < 0.05; **P < 0.01; ***p < 0.001 (Student's t test).

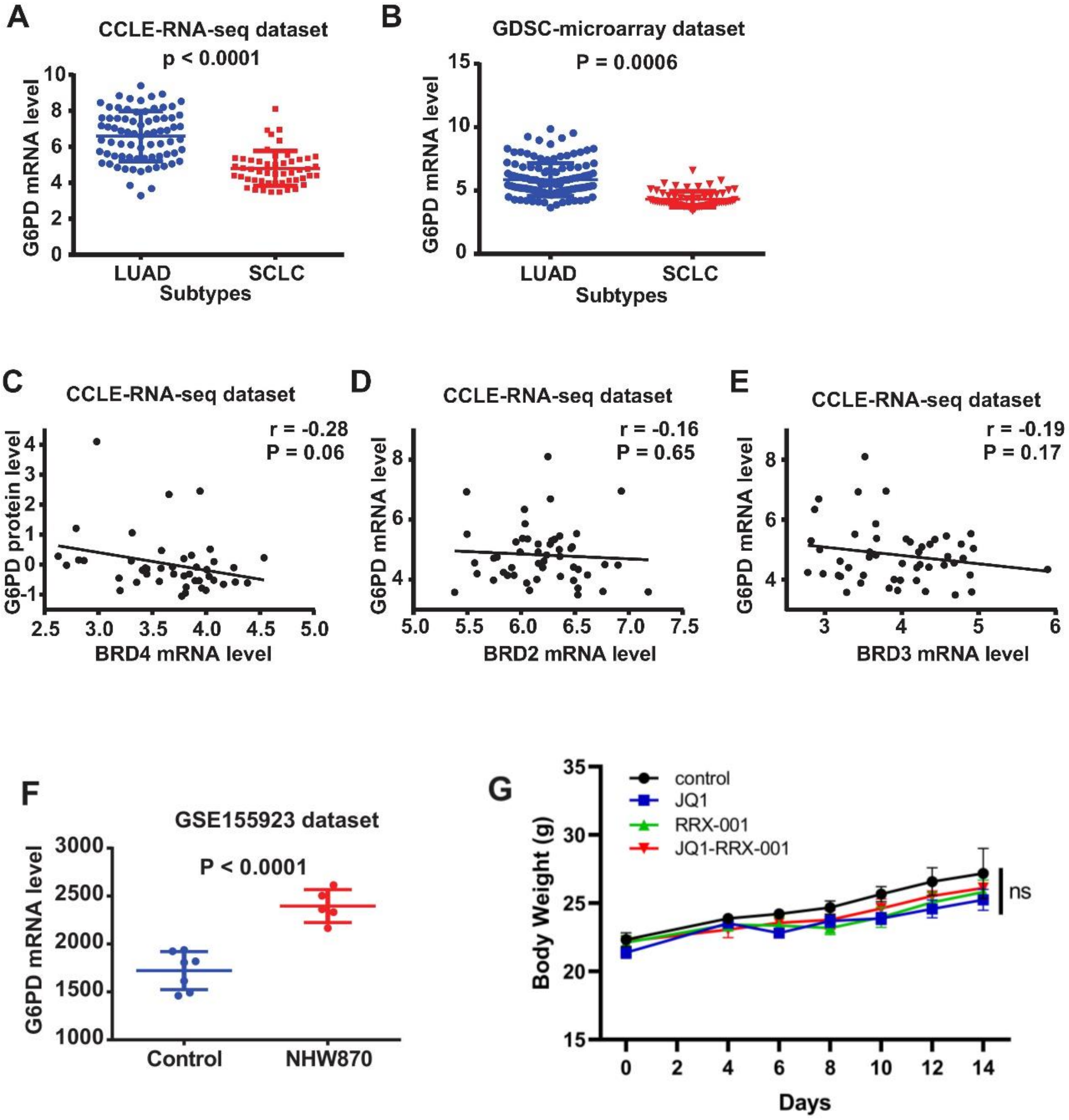

Supplementary Figure S4. Correlation between BRD4 and G6PD. A-B) Analysis of G6PD expression difference between LUAD and SCLC based on CCLE database (**A**) and GDSC database (**B**). (**C–E**) scatter diagrams show the correlation between BET family and G6PD expression in CCLE SCLC cell line data. (**F**) The effect of NHW870 treatment on G6PD expression in tumors was

analyzed based on GSE155923 database. (**G**) The body weight of mice changed during treatment. ns > 0.05.

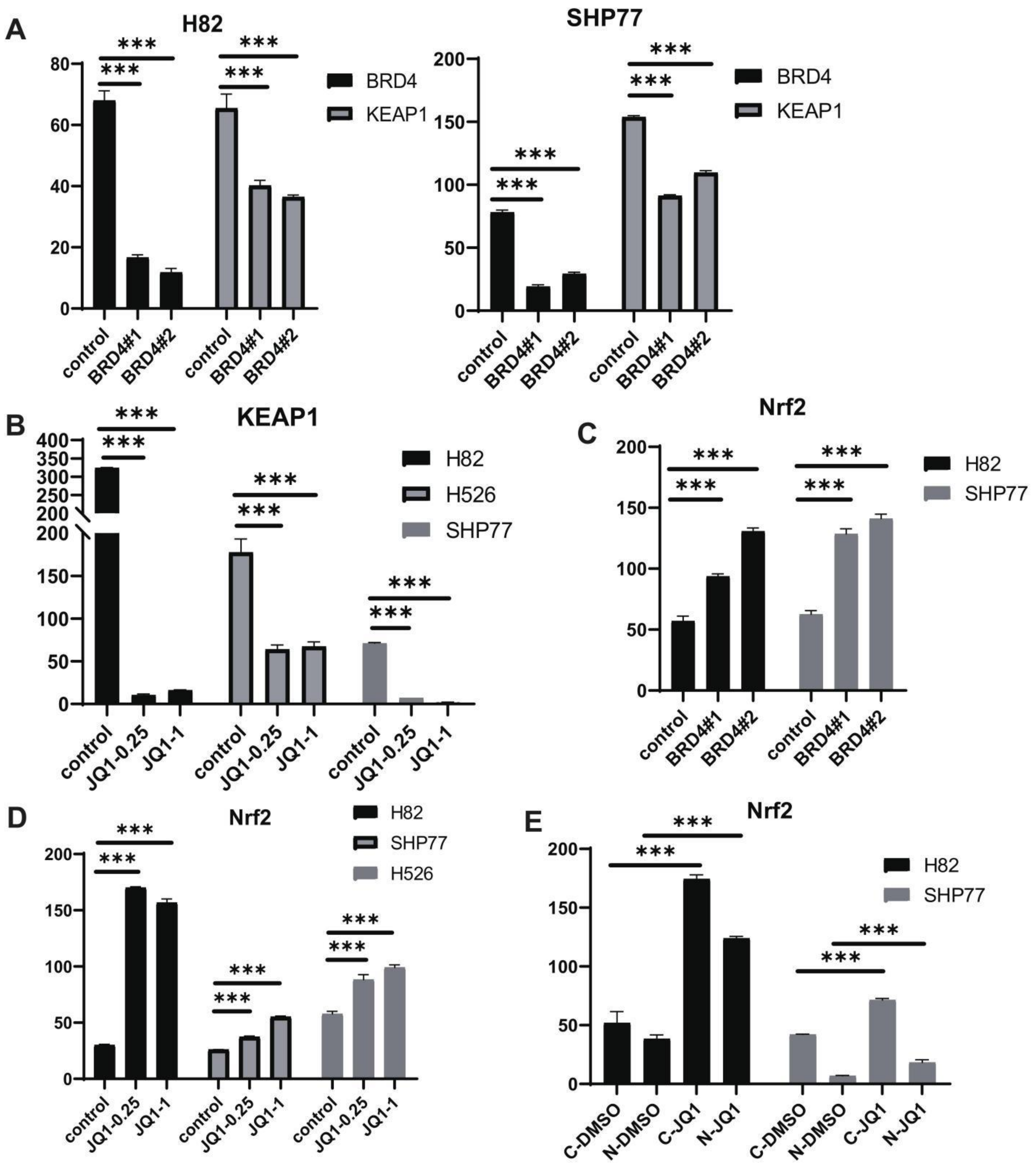

Supplementary Figure S5. The quantification data of figure 3 and figure 4. (**A**) showed densitometry analyses for BRD4, KEAP1 in figure 3B. (**B**) showed densitometry analyses for KEAP1 in figure 3E. (**C**) showed densitometry analyses for Nrf2 in figure 4A. (**D**) showed densitometry analyses for Nrf2 in figure 4B. (**E**) showed densitometry analyses for Nrf2 in figure 4C. Data are presented as the Mean ± SD, *P < 0.05; **P < 0.01; ***p < 0.001 (Student's t test).

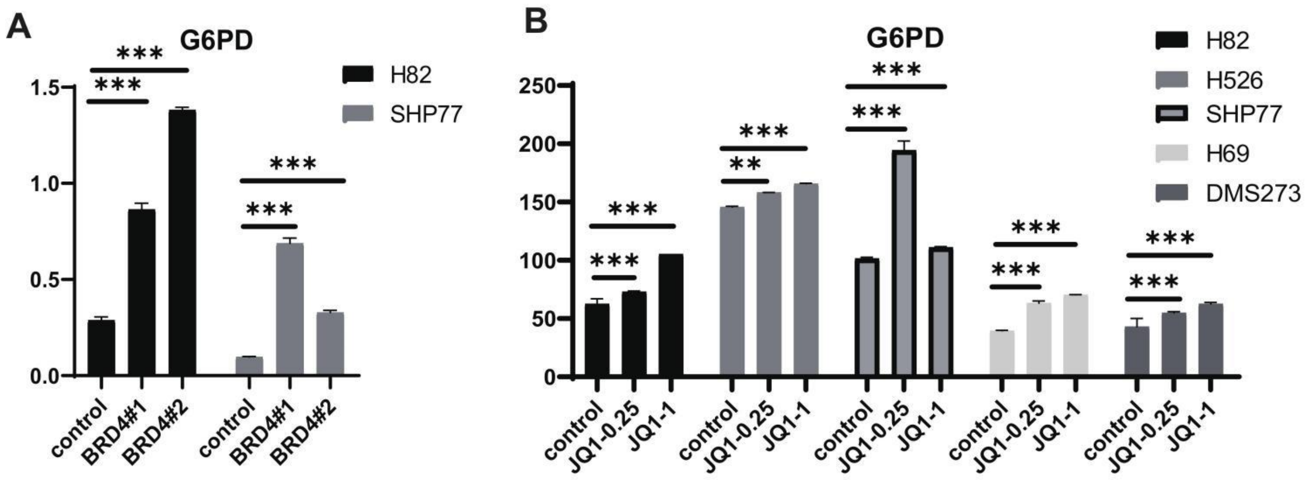

Supplementary Figure S6. The quantification data of figure 5. (**A**) showed densitometry analyses for G6PD in figure 5F. (**B**) showed densitometry analyses for G6PD in figure 5G. Data are presented as the Mean ± SD, *P < 0.05; **P < 0.01; ***p < 0.001 (Student's t test).

Supplementary Table S1.ROC analysis of KEAP1 expression for distinguishing cancers from normal tissues

Disease	Predictor variable	cut - off values(FPKM)	Sensitivity	Specificity	AUC
LUNG	KEAP1	4.343	0.935	0.622	0.785
BRCA	KEAP1	4.276	0.796	0.658	0.748
COAD	KEAP1	4.730	1.000	0.504	0.801
LIHC	KEAP1	3.910	0.920	0.873	0.922
PRAD	KEAP1	4.746	0.885	0.501	0.717
THCA	KEAP1	4.529	0.690	0.657	0.699
ESCA	KEAP1	3.699	0.636	0.870	0.747
READ	KEAP1	4.444	0.900	0.789	0.857
